# Impact of endoscopic metallic stent placement and emergency surgery on detection of viable circulating tumor cells for acute malignant left-sided colonic obstruction

**DOI:** 10.1186/s12957-022-02879-6

**Published:** 2023-01-02

**Authors:** Zhizhan Ni, Yuepeng Cao, Liming Liu, Chenshen Huang, Huahao Xie, Jinzhe Zhou, Bujun Ge, Qi Huang

**Affiliations:** 1 Department of General Surgery, Tongji Hospital, Tongji University School of Medicine, Shanghai, China; 2 Department of Colorectal Surgery, Ningbo First Hospital, Ningbo, China; 3 Department of General Surgery, Shanghai Jing’an Shibei Hospital, Shanghai, China; 4 Department of Gastrointestinal Surgery, Fujian Provincial Hospital, Fuzhou, China

**Keywords:** Circulating tumor cells, Acute malignant left-sided colonic obstruction, Colorectal cancer, Biomarker, Self-expanding metallic stent

## Abstract

**Background:**

Self-expanding metal stents (SEMS) served as a bridge to surgery (BTS). However, this method may be associated with worse long-term prognosis and relapse of CRC patients. Therefore, we attempted to clarify this in the angle of circulating tumor cells (CTCs).

**Methods:**

A multicenter study was performed from March 2018 to January 2021. Thirty-two colorectal cancer patients with obstruction were selected, of which 21 patients were performed SEMS as a BTS while 11 patients were performed emergency surgery. Bloods samples were collected in two groups of patients for further detecting CTCs. In the SEMS group, the samples were collected before and after stent insert and after radical surgery performed. In the ES group, the samples were collected before stent insert and after emergency surgery performed.

**Results:**

The number of CTCs did not show statistically significant differences before and after stent placement (34.90 vs 38.33, *p*=0.90), neither between the SEMS group and ES group in initial CTC levels (34.90 vs 58.09, *p*=0.394). No significant differences (38.33 vs 58.09, *p*=0.632) were observed after stent insert in the SMES group and the initial CTC levels in the ES group. Moreover, no major differences (24.17 vs 42.27, *p*=0.225) were observed after radical operation performed in both groups.

**Conclusion:**

The treatment of SEMS does not cause an increase in the number of CTC after stent insertion. Furthermore, there are may be other factors besides CTC to cause these poorer oncologic outcomes after SEMS placement.

## Introduction

Colorectal cancer (CRC) is one of the most commonly diagnosed cancer worldwide. Up to 30% of patients with CRC suffered acute colonic obstruction [1–3], at the time of the initial diagnosis [4]. Self-expanding metal stents (SEMS) serve as a bridge to surgery (BTS), which provide more opportunities to prepare for radical surgery. SEMS and conventional emergency surgery (ES) [3, 5, 6] have become two conventional treatments which widely utilized in malignant obstruction, especially left-sided colonic obstruction. However, the method of SEMS as a BTS may be associated with worse long-term prognosis and relapse of CRC patients.

Circulating tumor cells (CTCs) are potential substitutes for distant metastasis and promising novel biomarkers for tumors [7], which can gain access to the circulatory system and are detectable in the peripheral blood [8]. In our previous study [9], we found that compared with emergency surgery, SEMS are associated with more perineural invasion, a higher recurrence rate, and worse overall survival in patients. Nevertheless, the underlying mechanisms remain unresolved. Based on this, we doubt that CTCs were increased after SEMS placement in some way to influence the prognosis and recurrence.

In this study, we employed a novel method to capture CTCs with flow-cell system and analyzed the CTCs from a cohort of 32 colorectal cancer patients with obstruction. We examined the differences between the number of CTCs before and after the treatment of SEMS or ES in CRC patients to evaluate whether the treatment of SEMS will influence the CTC after stent insertion.

## Methods

This prospective multicenter study was conducted at Shanghai Tongji Hospital and Ningbo First Hospital. We examined differences in the numbers of CTCs before and after SEMS or ES. We included the patients with primary CRC with acute left-sided colonic obstruction from March 2018 to January 2021. The study protocol was approved by the institutional review board of Tongji Hospital Affiliated to Tongji University (approved no. KYSB-2017-007). The clinical trial was registered (ChiCTR-OON-17010877). Written consent was obtained from all the participants before study.

### Patient selection

Patients were considered to have acute colonic obstruction based on clinical signs of colonic obstruction (abdominal distention, constipation, and vomiting) and related radiological signs under computed tomography (CT) scan. Patients were then separated into 2 distinct groups based on the type of procedure performed. The SEMS group included patients who were underwent SEMS placement while the ES group included patients who were treated with emergency surgery or failed to insert stent and transferred to emergency surgery.

### Blood collection and CTC detection

Peripheral blood samples (5 ml) were collected into tubes containing ethylenediaminetetraacetic acid sodium (EDTA-NA). In the SEMS group, we collected blood samples for three times respectively, which denoted as first CTC for before stent placement, second CTC for 7 days after SEMS placement, and third CTC for7 days after radical surgery. In the ES group, we collected blood samples before and after emergency surgery, denoted as first and second CTC, respectively.

First, the blood samples were centrifuged and the supernatant discarded. Then, cells were then subjected to erythrocyte lysis and filtered prior to staining. The rest cells were then subjected to erythrocyte lysis and filtered prior to staining. After centrifugation, cells were washed and resuspend in PBS with 1%BSA and eliminate the majority of RBC and WBC by using the FlowCell® CTC enrichment system (Pola- ris Biology, Shanghai, China). Next, they were incubated for 40 min at 37°C with Cy5 conjugated anti-CD45 antibody. After that, 0.4 mM 2-deoxy-2-[(7-nitro-2,1,3-benzoxadiazol-4-yl)amino]-d-glucose (2-NBDG) and hoechst33342 at a concentration of 0.5 g/ml were used to incubated the cells for another 20 min. The cells centrifuged onto the glass after rinsing with PBS. Subsequently, the cells were scanned and imaged by EVOS FL Auto 2 (Invitrogen, MA, USA), a high-content screening system, in three fluorescent colors. The images and identified candidate tumor cells, analyzed by a computational algorithm, which were feasible and show high-glucose uptake and reviewed by experienced technicians. High-glucose uptake cells with high 2-NBDG intensity and negative CD45 staining were recognized as potential CTCs [10].

### Stent placement and surgery

All procedures were performed under general anesthesia. Stent placement was performed following standard protocols as follows [11]. A guide wire was passed through the stenosis and obstruction, and then, the stent was deployed on the guide wire under endoscopy. Confirm the correct positioning of the bracket by CT and endoscopy. The SEMS group suffered radical surgery after obstruction relieving. Both groups underwent standard colectomy and regional lymphadenectomy. The surgical approach, surgical method, and resection range are determined by the surgeon according to the tumor location, tumor stage, and general conditions of the patient.

### Statistical analysis

All statistical analyses were performed with R software version 4.2.1. *χ*^2^ tests or Fisher’s exact tests were used for categorical variables. Student’s *t* test for paired-sample *t* test was used to analyze paired differences.

## Results

### Baseline characteristics

**Fig. 1:**
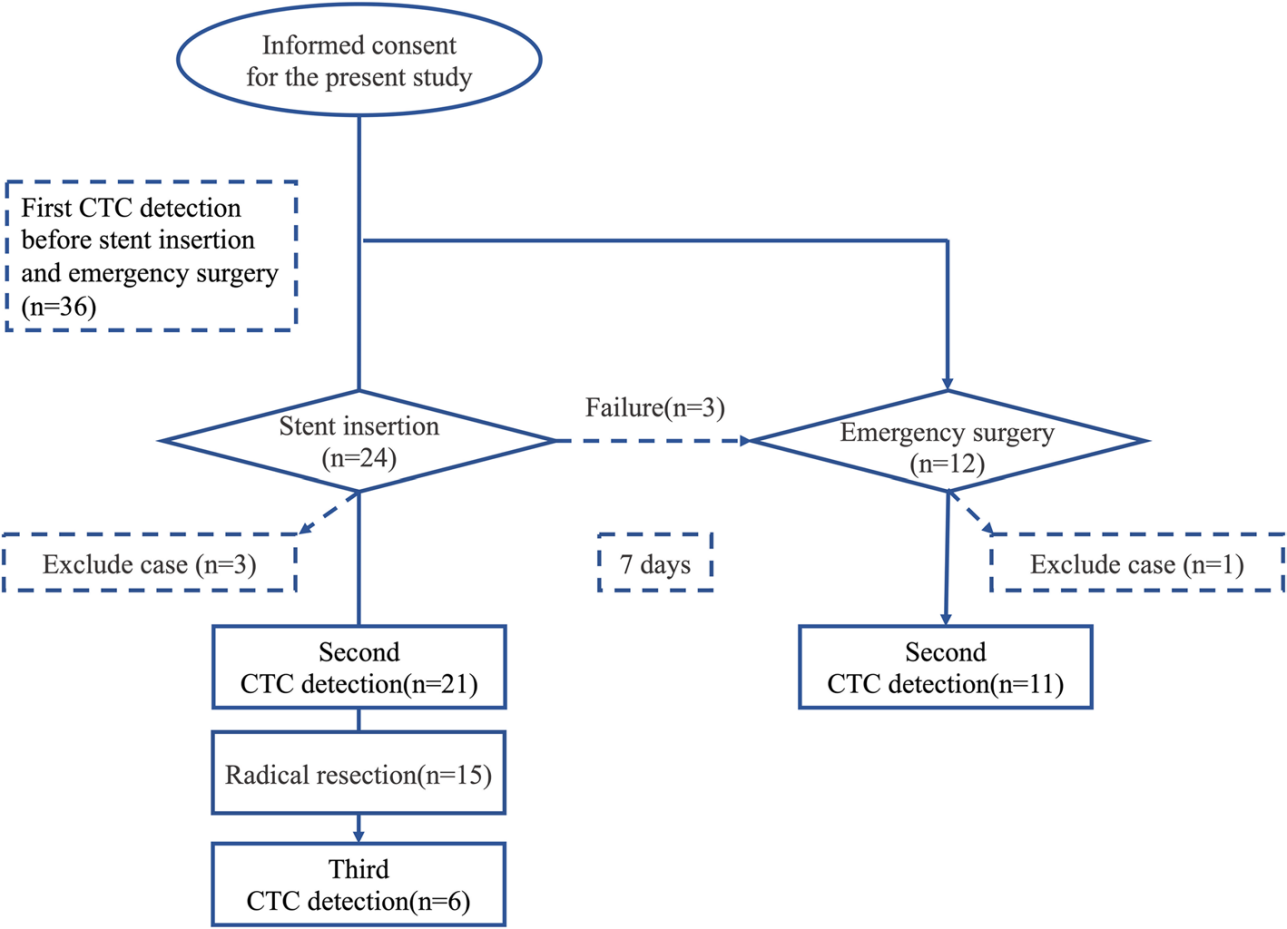
Flowchart of patient selection

**Fig. 2:**
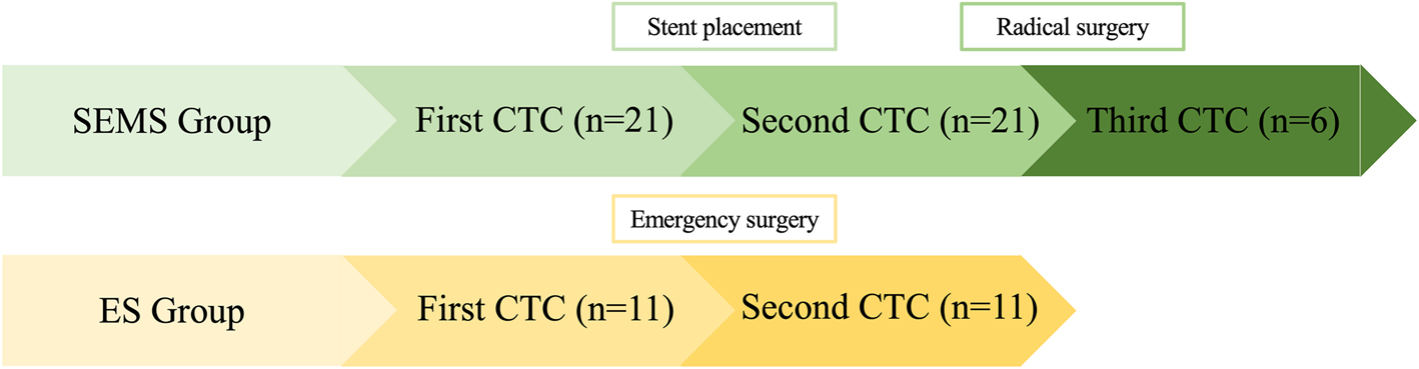
The flowchart of collect blood samples and stent insertion

From March 2018 to January 2021, 36 patients were enrolled. A flowchart of these patients is shown in Fig. 1. After exclusion of patients with palliative operation (*n*=3) and patient occurred myocardial infarction (*n*=1), a total of 32 patients were included in the subsequent analysis (Fig. 2). Stent insertion was performed in 24 patients, 3 of which underwent emergent surgery owing to stent placement failure and then transferred to the ES group. Emergency surgery was performed in 12 patients in all while one patient was excluded owing to occurred myocardial infarction on the first postoperative day. In the SEMS group, 15 patients underwent radical surgery 7 days (5–8 days) after stent placement, and 6 patients underwent palliative surgery. CTC detection was performed before operative procedures and after 7 days of operative procedures which include stent placement, ES operation, and operation after stent placement.

In the SEMS group (Table 1), the average age was 71.24 (8.9) year [mean (SD), years]. Obstructive CRC were located in the transverse colon (left-side) in 2 patients, descending colon in 4 patients, and sigmoid colon in 15 patients. Among them, 9 patients were diagnosed as cTNM stage II and 12 patients were diagnosed as cTNM stage III.

**Table 1:** Baseline characteristics of the SEMS group

		SEMS (*n*=21)
Age (mean (SD), y)		71.24 (8.9)
	Male	13
	Female	8
Technical success		21/24
Clinical success		20/21
Overall complications		
	Perforation	1
	Migration	0
	Re-obstruction	0
Primary tumor site		
	Transverse colon	2
	Descending colon	4
	Sigmoid colon	15
cTNM stage		
	II	9
	III	12

Postoperative clinical data (Table 2) include surgical approach, the permanent stoma rate, adjuvant therapy, and postoperative TNM stage. Seven of 11 patients suffered permanent stoma in the ES group while 3 of 15 patients suffered in the SEMS group who underwent radical surgery (*p*=0.043). Thirteen of 15 patients in the SEMS group underwent laparoscopic resection compared 2 of 11 patients in the ES group (*p*=0.001). Seven of 11 patients received adjuvant therapy while 14 patients in SEMS (*p*=0.128). Other postoperative characteristics, including pT stage (*p*=0.999), pN stage (*p*=0.398), and pM stage (*p*=0.999).

**Table 2:** Postoperative clinical data

		SEMS (*n*=15)	ES (*n*=11)	*P* value
Age (mean (SD), y)		71.33 (10.1)	70.9 (8.9)	0.769
Sex				0.628
	Male	9	8	
	Female	6	3	
Primary tumor site				0.669
	Transverse colon	2	3	
	Descending colon	3	2	
	Sigmoid colon	10	6	
pT stage				0.999
	T3	3	2	
	T4	12	9	
pN stage				0.398
	N0	8	3	
	N1	3	4	
	N2	4	4	
pM stage				0.999
	M0 stage	14	10	
	M1 stage	1	1	
Surgical approach				0.001
	Laparotomy	2	9	
	Laparoscopy	13	2	
Primary resection type				0.043
	Without stoma	12	4	
	With stoma	3	7	
Adjuvant therapy		14	7	0.128

### Technical success of SEMS placements

Twenty-four patients underwent SEMS insertion; however, the guide wire failed to pass through the stenosis and obstruction in 3 patients. All of 3 patients transferred to the ES group and performed conventional emergency surgery to decompress.

### Clinical success of relieving obstruction and complications

Among the 21 patients in the SEMS group, 1 patient suffered perforation 8 days after SEMS placements and underwent surgery promptly. In the ES group, 12 patients underwent emergency surgery successfully, and only one patient occurred myocardial infarction on the first postoperative day and died on the fifth day (excluded case).

### Detection of CTCs

**Fig. 3:**
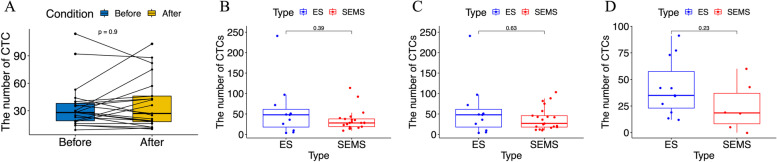
**A** The number of CTCs before/after stent placement (*p*=0.90). **B** The number of CTCs before stent placement and before ES (*p*=0.39). **C** The number of CTCs after stent placement and before ES (*p*=0.63). **C** The number of CTCs after radical surgery in the SEMS group and after ES (*p*=0.23)

The number of CTCs detected within the peripheral blood circulation before/after SEMS placement and operation or ES in 7 days. As shown in Fig. 3 A, B, there were no statistically significant differences before and after stent placement (34.90 vs 38.33, *p*=0.90), neither between the SEMS group and ES group in the first CTC levels (34.90 vs 58.09, *p*=0.394). We further compared the second CTC in the SEMS group and first CTC in the ES group, no significant differences (38.33 vs 58.09, *p*=0.632) were observed (Fig. 3C). In order to analysis whether ES or operation after stent placement would have effects on the number of CTCs, we compared the third CTC in SEMS and the second CTC in ES; however, no major differences (24.17 vs 42.27, *p*=0.225) were observed in two groups (Fig. 3D).

We then divided SEMS patients into three groups according to the CTC variations before and after stent insert, which are increased, decreased, and not change groups. Then, we analyzed these three groups in different dimensions, as shown in Table 3. Among these 21 patients, 11 patients decreased, 9 patients increased, and 1 patient did not change. However, there are no difference in age (*p*=0.793), sex (*p*=0.098) primary tumor site (*p*=0.327), and cTNM stages (*p*=0.465). We further analyzed the CTC variations before stent insert and after operation. Among them, 3 patients increased and 3 patients decreased (Table 4). There is also no difference in age (*p*=0.104), sex (*p*=0.999), primary tumor site (*p*=0.513), and pT stages (*p*=0.999). Finally, we did this kind of analysis in the ES group (Table 5). There are only 2 patients increased in the ES group, while 9 decreased. There is no difference in age (*p*=0.283), sex (*p*=0.425), primary tumor site (*p*=0.361), and cTNM stages (*p*=0.425).

**Table 3:** The clinical comparison between the increased, decreased, and not changed groups in the SEMS group before and after stent insert

		SEMS (*n*=21)			
		Increased (*n*=9)	Decreased (*n*=11)	Not changed (*n*=1)	*P* value
Age (mean (SD), y)		72.11 (11.74)	70.55 (6.92)	70	0.793
Sex					0.098
	Male	9	4	0	
	Female	2	5	1	
Primary tumor site					0.327
	Transverse colon	1	2	1	
	Descending colon	1	1	0	
	Sigmoid colon	7	8	0	
cTNM stage					0.465
	II	4	4	1	
	III	5	7	0

**Table 4:** The clinical comparation between the increased and decreased groups in the SEMS group before stent insert and after operation

		SEMS (*n*=6)		
		Increased (*n*=3)	Decreased (*n*=3)	*P* value
Age (mean (SD), y)		75.00 (4.583)	60.00 (11.53)	0.104
Sex				0.999
	Male	2	1	
	Female	1	2	
Primary tumor site				0.513
	Transverse colon	0	1	
	Descending colon	1	1	
	Sigmoid colon	1	1	
pT stage				0.999
	T3	0	0	
	T4	3	3	
pN stage				0.999
	N0	3	2	
	N1	0	0	
	N2	0	1	
pM stage				0.999
	M0	3	3	
	M1	0	0

**Table 5:** The clinical comparation between the increased and decreased groups in the ES group before stent insert and after operation

		ES (*n*=11)		*P* value
		Increased (*n*=2)	Decreased (*n*=9)	
Age (mean (SD), y)		64.50 (7.78)	72.33 (8.90)	0.283
Sex				0.425
	Male	1	7	
	Female	1	2	
Primary tumor site				0.361
	Transverse colon	0	3	
	Descending colon	1	1	
	Sigmoid colon	7	8	
cTNM stage				0.425
	II	1	2	
	III	1	7	
pT stage				0.197
	T3	1	1	
	T4	1	8	
pN stage				0.478
	N0	1	2	
	N1	0	4	
	N2	1	3	
pM stage				0.999
	M0	2	8	
	M1	0	1	

## Discussion

This study is a multicenter trial that analyzed whether SEMS placement would cause an increase in the number of CTC. We employed a novel method to capture CTCs in 32 patients, 21 of these were treated with SEMS, and 11 were treated with ES. The results suggest that CTC counts obtained before and after placement did not differ significantly. Furthermore, we analyzed the CTC counts in the SEMS and ES group in different stages, but no statistical difference was observed also.

Since the concept of CTC came out, it has been considered as a poor prognostic characteristics and poor survival outcome [12]. Yang et al. found that [13, 14], for CTC-positive patients with CRC, despite of the sampling time, adjuvant therapy, and TNM stage, they revealed a shorter overall survival and disease-free survival in their meta-analysis. However, several scholars considered CTCs detected at different follow-up time points after surgery were of different prognostic impact [15] and the methods of detecting CTCs will influence the overcome [16]. Racila and colleagues [17] first described a novel method to detect the extremely rare CTCs in 1998, which is the gold standard nowadays [18]. They used an immunomagnetic enrichment technique to detect CTCs which based on epithelial cell adhesion molecule (EpCAM) [19]. Another important method to detect CTC is based on nucleic acid identification by PCR methods [20–22]. Here, we used a microfluidic device to detect CTC based on crossflow filtration technology, then distinguishes CTCs by analyzing glucose uptake of fluorescent-labeled 2-NBDG [10]. This method based on 2-NBDG staining to identify metabolically active cells in blood; however, whether to claim that all 2-NBDG-positive cells are cancer cells is reasonable remains further exploration. Lu et al. also employed this method to detect CTC from lung cancer patient and concluded that CTCs can be served as a biomarker to assist the diagnosis and predict lung cancer metastasis [10]. Inclusive analysis these results, we found that this method could indeed help to explore more CTCs compared with other methods.

To date, some studies have demonstrated that stent insertion may result in tumor cell dissemination into the peripheral circulation and may induce distant metastases [16]. Nesteruk confirmed that CTC detection 7 days after surgery was of prognostic significance for the local recurrence, while 24 h not [23]. Shinya et al. [24] also found that an increase in the number of CTC after stent insertion and that the pressure exerted on the tumor during SEMS expansion result in a direct release of some cancer cell, which will induce recurrence despite R0 resection or adjuvant therapy. In our study, we did also collect blood samples 7 days after surgery. However, the CTC counts before and after surgery in the SEMS and ES groups did not show significant difference. Thus, we conclude that stent insertion may not result tumor cell dissemination and increase the CTC number. Thus, combined with previous study findings and other studies, we speculated that there are also other factors affecting patients’ prognosis, such as peripheral nerve invasion [25–27] and the alteration of epigenetic [28].

The present study had several limitations. First, the study was a non-randomized study, so selection bias may exist in the choice of decompression method. Second, the volumes of clinical samples were limited and we did not carry out survival analysis due to immature survival data. Third, ctDNA may be both more sensitive and more accurately [29], which is widely used in clinic. In hence, ctDNA will be considered for later research.

## Conclusion

In conclusion, our analysis of CTC counts which captured by the novel method of FlowCell system in this cohort of 32 patients suggested that the treatment of SEMS does not cause an increase in the number of CTC after stent insertion. Furthermore, there are may be other factors besides CTC to cause these poorer oncologic outcomes after SEMS placement.

## Data Availability

Not applicable.

## Abbreviations

SEMS: Self-expanding metal stents
BTS: Bridge to surgery
CTCs: Circulating tumor cells
CRC: Colorectal cancer
CT: Computed tomography
EDTA-NA: Ethylenediaminetetraacetic acid sodium
2-NBDG: 2-Deoxy-2-[(7-nitro-2,1,3-benzoxadiazol-4-yl)amino]-d-glucose
EpCAM: Epithelial cell adhesion molecule
ctDNA: Circulating tumor DNA
